# Patients potentially in need for palliative care in Germany—A regional small-area estimation based on death registry data

**DOI:** 10.1007/s43999-025-00070-4

**Published:** 2025-07-11

**Authors:** Daniela Gesell, Farina Hodiamont, Claudia Bausewein, Eva Grill, Daniela Koller

**Affiliations:** 1 Institute of Medical Data Processing, Biometrics and Epidemiology (IBE), Faculty of Medicine, LMU Munich, Munich, Germany; 2 Department of Palliative Medicine, LMU University Hospital, LMU Munich, Munich, Germany; 3 German Center for Vertigo and Balance Disorders (DSGZ), LMU University Hospital, LMU Munich, Munich, Germany

**Keywords:** Palliative care, Palliative care need, Death registry data, Regional analysis, End-of-life care

## Abstract

**Background:**

Demographic change and the increasing prevalence of chronic illnesses lead to a higher demand for palliative care. Currently, little is known about potential need for palliative care at a small-area level in Germany. However, this is crucial for the planning of services. We aimed to calculate the proportion of the population potentially in need of palliative care on a small-area level and to illustrate the nationwide variations.

**Methods:**

Retrospective cross-sectional study based on causes of death statistics in Germany. Causes of death of all adult deceased in Germany in 2022 were included. The potential need for palliative care was identified based on Murtagh et al. (2014) using ICD-10-codes. Geographic variation was analyzed on district level.

**Results:**

1,062,452 persons were documented in the causes of death statistics, of which 752,643 died with a potential palliative care need (70.8%). Overall mean age was 79.5 years (SD 12.7), 50.1% were female (*n* = 532,248). Most deaths were due to neoplasms (23,675; 22.6%) and cardiovascular diseases (230,338, 21.7%). The numbers of deceased with potential need per 100,000 inhabitants varied between districts from 578 to 1,438, with highest values in districts in Saxony, Thuringia, Saxony-Anhalt, and lowest in Bavaria and Baden-Wuerttemberg.

**Discussion:**

Our definition of potential palliative care need included both deaths due to oncological diseases, who commonly receive palliative care, and non-oncological conditions. The findings highlight the regional differences in potential palliative care need on small-area level and the importance of comprehensive healthcare planning adapted to the specific needs of individuals.

## Background

Demographic change and the growing number of patients with chronic diseases increase the need for palliative care in Germany. According to the World Health Organization palliative care is ‘a crucial part of integrated, people-centered health services. Relieving serious health-related suffering, be it physical, psychological, social, or spiritual, is a global ethical responsibility.’ [1] It is essential that palliative care is available to alleviate suffering in all healthcare sectors, irrespective of the underlying cause of disease, whether cardiovascular disease, cancer, organ failure or frailty of old age. As a consequence, the World Health Organization estimates that 56.8 Mio people worldwide are in need of palliative care annually [1]. 

Generally, the provision of palliative care is divided into general palliative care, provided by general practitioners, and specialist palliative care, provided by multi-professional teams specialized in palliative care [2]. The choice of the appropriate palliative care provision is dependent on the individual complexity of the patient and their care situation [3]. Due to this variation it is challenging to estimate palliative care need on regional or national levels [4]. The absence of a clear definition of palliative care need impedes accurate calculation of the potential need. The number and underlying causes of deaths derived from administrative data can serve as an initial reference point for estimating palliative care need within a population [5]. It is hypothesized that all individuals who did not experience a sudden or trauma/injury-related death may have benefitted from palliative care support [6, 7]. 

Using different definitions of need and inclusion criteria, estimates for potential palliative care need based on administrative data varied from 50 to 89% of deceased persons of a given population [7–9]. These definitions included disease-specific mortality data and prevalence of symptoms such as pain, dyspnea or depression [10]. In other definitions, selected causes of death and linked hospital admissions data were used, including all deaths that occurred in hospital with the same condition as stated on the death certificate [7], or percentage of deaths plus chronic disease data derived from a study conducted in Spain [11]. A refined analysis of Murtagh et al. also addressed the respective palliative care relevant diagnosis codes for changes in diagnostic and treatment practices [4]. They estimated that 63% of all deceased persons would have had a potential need for palliative care before their death [4]. 

For the German context, Scholten et al. calculated a range of estimations of how many people may be in need of palliative care [12]. They described that between 40.7% and 96.1% of the deceased population in Germany would have potentially needed palliative care, with 80.3% of palliative-sensitive cases in the deceased over 80 years [12]. The methods outlined can be applied in different countries where ICD-10-coded death registration data are available. The small-area perspective of death registrations can provide further valuable insights into potential palliative care need, as specific causes of death indicate potential palliative care requirements. These insights allow for comparisons between estimated need and the current level of provision [4]. The need for palliative care can vary depending on regional socio-demographic and geographical conditions, making it crucial to examine these differences on a regional level. Yet, death registrations have been used for place-of-deaths-analyses on a small-area level [13–15], only one Canadian study addressed small-area variation in palliative care [9]. As to date, detailed estimates on palliative care need at a small-area level in Germany are not available. This limits the ability to address region-specific disparities. Therefore, our aim was to calculate the proportion of the population potentially in need of palliative care on a small-area level and to illustrate the nationwide variations.

## Methods

### Study design

We conducted a retrospective study with data of the Research Data Centres of official statistics (FDZ) following the RECORD (REporting of studies Conducted using Observational Routinely-collected health Data) statement [16]. The study was reviewed by the Local Research Ethics Committee of Ludwig-Maximilians-University Munich and a waiver has been granted (reference number 24–0533 KB).

### Setting and data sources

We analyzed official cause of death statistics in Germany from the year 2022. Germany has 16 federal states and 400 districts and independent cities [17]. For this analysis, we used aggregated data on district level. In the year 2022, the population of Germany was estimated to be approximately 84 million [18]. 

The cause of death statistics is the elementary data base determining important health indicators such as mortality rates, years of life lost and avoidable deaths and provides information on the causes of death in the population. The statistics are a complete data base of all deaths in Germany. A distinction is made between the conditions that led directly and indirectly to death. Using the ICD-10 classification, they identify the underlying disease, defined as the primary cause of death that is considered to have initiated the sequence of events leading to death [19]. According to the definition of the World Health Organization this is „the disease or injury which initiated the train of morbid events leading directly to death, or the circumstances of the accident or violence which produced the fatal injury“ [20]. It is important to note that death certificates, when completed in full and in accordance with the specified guidelines, serve as a fundamental source of data for mortality analyses. Such analyses can contribute to the enhancement of healthcare services. Death certificates can be edited by coding specialists or by the IRIS/Muse software in the statistical offices [21]. This is an automated system that is designed for the coding of multiple causes of death and the identification of the underlying cause of death [22]. The standardized methodological approach and the continuity of the data base ensure that these data are reliably available for scientific research [19]. The socio-demographic characteristics of all deceased individuals and their cause of death are collected annually. The data is spatially differentiated down to the level of districts and independent cities [23]. The cause of death statistics are based on the 10th revision of the of the “International Statistical Classification of Diseases and Related Health Problems” (ICD-10-WHO) [24]. 

The database provided by the Federal Statistical Office does not contain any personal data. The anonymized data can be accessed upon request for specific research questions using a secured analysis environment.

### Data Preparation and categorization

Given the data constraints in Germany and the difficulty of aligning mortality data with hospital data on symptom prevalence or disease, we used the more detailed breakdown of palliative care relevant ICD-10 diagnoses for inclusion or exclusion used by Murtagh et al. [4] The codes defined as potential palliative care need are displayed in Table 1. Two distinct groups have been calculated, one comprising cases where the defined codes apply (deceased with potential palliative care need) and the other comprising cases where the codes do not apply (deceased without potential palliative care need).

**Table 1:** Categorization of ICD-10-WHO codes for potential palliative care need

ICD-10 Code	Label
C00-C97	Malignant neoplasms
F01, F03, G30, R54	Alzheimer’s, dementia and senility
G10, G20, G35, G122, G903, G231	Neurodegenerative disease
I00-I52, I60-I69	Heart disease, including cerebrovascular disease
J06-J18, J20-J22, J40-J47, J96	Respiratory disease
K70-K77	Liver disease
N17, N18, N28, I12, I13	Renal disease
B20-B24	HIV/AIDS

### Statistical analysis

Data was analyzed and displayed on a regional level. Descriptive statistics were used to aggregate deceased individuals per district. In an overall death-certificate approach, the percentage of deceased individuals with potential palliative care need was calculated. This was complemented by a sub-group analysis of those who died with cancer. A Chi-square test of independence was conducted to examine the association between gender and the potential need for palliative care, with the Phi coefficient used to measure the strength of the association. A t-test for independent samples was conducted to compare the average age at death between individuals who have had a potential palliative care need and those who did not.

To illustrate regional differences in potential palliative care need, it is essential to consider the population distribution of each region. Therefore, a population-based approach was conducted, which accounted for population distribution by calculating the number of deaths with potential palliative care need per 100,000 inhabitants. Furthermore, the proportion of deceased individuals who died from an oncological disease was calculated at district level, to compare these results with the potential need for palliative care. Choropleth maps on district level were constructed to illustrate regional disparities, employing Jenks natural breaks to segment the data into categories with lower values in light colors to higher values in dark colors.

All cartography and analyses were conducted using ESRI ArcGIS Pro (2.8) and IBM SPSS (29).

## Results

### Cohort and overall potential palliative care need

A total of 1,062,452 people died in Germany in 2022, 752,643 of which had a potential palliative care need. This represents 70.8% of all deaths in Germany or approximately 892 persons per 100,000 of the population per year. The mean age of all deceased was 79.5 years (SD 12.7 years), with 50.1% being female (*n* = 532,248) (Table 2). The t-test showed a statistically significant difference in the mean age at death between individuals with potential palliative care need (M = 80.1 years) and those without (M = 77.9 years), t(489,463) = -76.1, *p* < 0.001. Specifically, individuals who died with potential palliative care need were older, by a mean of 2.2 years (CI 95% -2.28 to -2.17). From all deceased, 72.5% of women and 69.1% of men had a potential need for palliative care (χ2(1) = 1477.119, *p* < 0.001), with a Phi coefficient (ϕ = 0.037) indicating a weak association.

**Table 2:** Characteristics of deceased in Germany

	Total	Without potential palliative care need	With potential palliative care need	*p*-value
Total (%)	1,062,452 (100.0)	309,809 (29.2)	752,643 (70.8)	
Age, years mean ± SD	79.5 ± 12.7	77.9 ± 14.4	80.1 ± 11.8	< 0.001
Gender, n (%)				< 0.001
Female	532,248 (50.1)	146,200 (27.5)	386,048 (72.5)	
Male	530,204 (49.9)	163,609 (30.9)	366,595 (69.1)	

Table 3 shows the frequencies of causes of death stratified by potential palliative care need. Diseases of the circulatory system had the highest percentage of palliative care need, accounting for a third of all deaths (358,102), of which 96% having had a potential palliative care need. Neoplasms represented the second most frequent cause of death, accounting for 22% of deaths. Almost one-third of the deceased in the group without potential palliative care need died of injuries, poisoning and certain other consequences of external causes (15.3%) and with symptoms, signs and abnormal clinical and laboratory findings, not elsewhere classified (12.3%) (Supplementary Table 1).

**Table 3:** Frequencies of causes of death in Germany (*N* = 1,062,452)

ICD Group	Description	Total		Without potential palliative care need		With potential palliative care need	
		n	%	n	%	n	%
**Total**		**1**,**062**,**452**	**100.0**	**309**,**809**	**29.2**	**752**,**643**	**70.8**
A00-B99	Infectious and parasitic diseases	17,288	1.6	17,024	98.5	264	1.5
C00-D48	Neoplasms	239,675	22.6	8,400	3.5	231,275	96.5
D50-D90	Diseases of the blood and blood-forming organs and certain disorders involving the immune mechanism	5,165	0.5	5,165	100.0	0	0.0
E00-E90	Endocrine, nutritional and metabolic diseases	40,466	3.8	40,466	100.0	0	0.0
F00-F99	Mental and behavioral disorders	68,767	6.5	9,509	13.8	59,258	86.2
G00-G99	Diseases of the nervous system	38,985	3.7	10,891	27.9	28,094	72.1
H00-H59	Diseases of the eye and adnexa	35	0	35	100.0	0	0.0
H60-H95	Diseases of the ear and mastoid process	49	0	49	100.0	0	0.0
I00-I99	Diseases of the circulatory system	358,102	33.7	13,867	3.9	344,235	96.1
J00-J99	Diseases of the respiratory system	67,566	6.4	11,382	16.8	56,184	83.2
K00-K93	Diseases of the digestive system	45,993	4.3	28,737	62.5	17,256	37.5
L00-L99	Diseases of the skin and subcutaneous tissue	1,955	0.2	1,955	100.0	0	0.0
M00-M99	Diseases of the musculoskeletal system and connective tissue	6,811	0.6	6,811	100.0	0	0.0
N00-N99	Diseases of the genitourinary system	28,933	2.7	15,457	53.4	13,476	46.6
O00-O99	Pregnancy, childbirth and the puerperium	30	0	30	100.0	0	0.0
P00-P96	Certain conditions originating in the perinatal period	121	0	121	100.0	0	0.0
Q00-Q99	Congenital malformations, deformations and chromosomal abnormalities	1,392	0.1	1,392	100.0	0	0.0
R00-R99	Symptoms, signs and abnormal clinical and laboratory findings, not elsewhere classified	40,703	3.8	38,102	93.6	2,601	6.4
S00-T98	Injury, poisoning and certain other consequences of external causes	47,500	4.5	47,500	100.0	0	0.0
U00-U99	Codes for special purposes	52,916	5.0	52,916	100.0	0	0.0

Concentrating on the group with potential palliative care need, Table 4 displays more detailed information on specific conditions according to the definition mentioned in the methods section. Cardiovascular diseases (43.5%) and cancer (30.7%) represent the majority of deaths. Neurological causes of death such as dementia or neurodegenerative diseases, and respiratory, liver and renal diseases represent a smaller share of death conditions.

**Table 4:** Number of deceased in Germany by cause of death potentially in need for palliative care

ICD-10 Code	Label	*n*	%
C00-C97	Malignant neoplasms	231,275	30.7
F01, F03, G30, R54	Alzheimer’s, dementia and senility	72,190	9.6
G10, G20, G35, G122, G903, G231	Neurodegenerative disease	17,763	2.4
I00-I52, I60-I69	Heart disease, including cerebrovascular disease	327,691	43.5
J06-J18, J20-J22, J40-J47, J96	Respiratory disease	56,184	7.5
K70-K77	Liver disease	17,256	2.3
N17, N18, N28, I12, I13	Renal disease	30,020	4.0
B20-B24	HIV/AIDS	264	0.0
**Total**		**752**,**643**	**100.0**

### Regional analysis: population-based approach

**Fig. 1:**
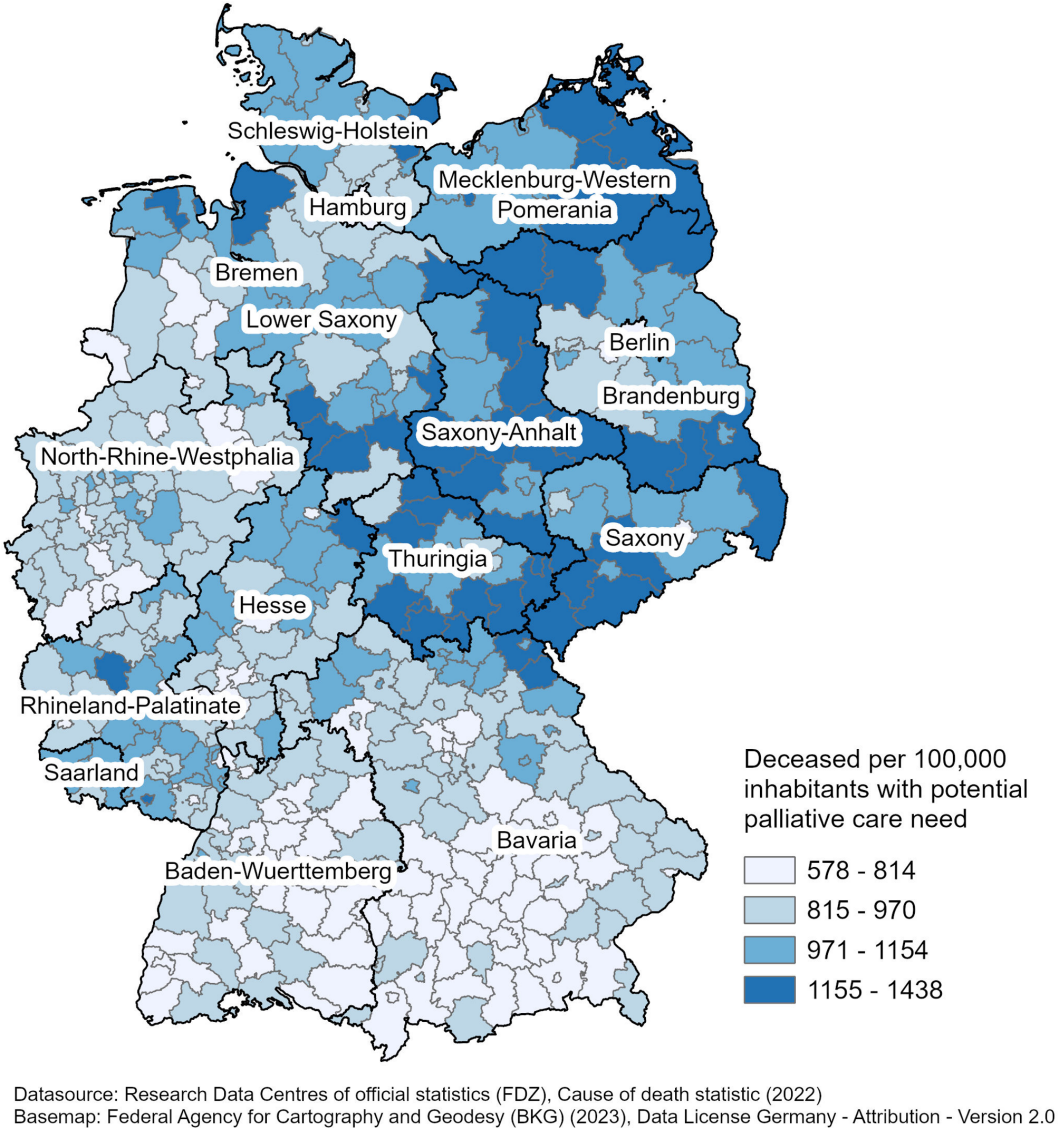
Deceased per 100,000 inhabitants with potential palliative care need

The analysis of potential palliative care need at district level revealed geographic variation. To account for the different inhabitants per region, a population-based approach is depicted. A map illustrating the number of deceased with potential palliative care need per 100,000 inhabitants across different districts is presented in Fig. 1. The map displays the distribution of deceased individuals per 100,000 inhabitants with a potential need for palliative care across Germany. The values range from 578 to 1,438 deceased with potential palliative care need per 100,000 inhabitants. The districts with the highest values, between 1,154 and 1,438 deceased with potential palliative care need per 100,000 inhabitants, are primarily located in the eastern part of the country, including Saxony, Thuringia, and especially some districts in Saxony-Anhalt, such as Mansfeld-Südharz, Salzlandkreis, and Harz, with more than 1,400 deceased per 100,000 inhabitants. The intermediate values, which range from 814 to 1,154 deceased with potential palliative care need per 100,000 inhabitants, are distributed across central and north-eastern regions. Lower values, ranging from 578 to 814 deceased with potential palliative care need per 100,000 inhabitants, can mainly be observed in western and southern German regions, including Bavaria, Baden-Wuerttemberg, and parts of North Rhine-Westphalia.

Table 5 presents the aggregated numbers of deceased individuals with potential palliative care need per 100,000 inhabitants across the federal states. The numbers show variation, with Saxony-Anhalt recording the highest numbers with 1,260 deceased with potential palliative care need per 100,000 inhabitants, followed by Mecklenburg-Western Pomerania (1,156/100,000), and Saxony (1,096/100,000). These states are predominantly located in eastern Germany. In contrast, the lowest numbers of deceased with potential palliative care need per 100,000 inhabitants were found in Hamburg (672/100,000), Berlin (737/100,000), and Baden-Wuerttemberg (775/100,000). The larger states in terms of population, such as North Rhine-Westphalia and Bavaria, had intermediate numbers of deceased individuals with potential palliative care need, with 864 and 803 per 100,000 inhabitants, respectively.

**Table 5:** Deceased with potential palliative care need per federal state

Federal states	*n* (deceased with potential palliative care need)	mean (%; deceased with potential palliative care need)	Deceased per 100.000 inhabitants with potential palliative care need
Baden-Wuerttemberg	87,439	70.4	775
Bavaria	107,407	70.7	803
Berlin	27,674	70.2	737
Brandenburg	26,976	72.3	1,048
Bremen	6,217	70.3	908
Hamburg	12,724	64.3	672
Hesse	54,196	72.2	864
Mecklenburg-Western Pomerania	18,827	74.4	1,156
Lower Saxony	80,513	74.5	989
North Rhine-Westphalia	156,718	67.2	864
Rhineland-Palatinate	38,868	72.3	935
Saarland	10,202	66.4	1,028
Saxony	44,769	74.7	1,096
Saxony-Anhalt	27,542	74.1	1,260
Schleswig-Holstein	29,099	71.7	985
Thuringia	23,472	71.8	1,104
**Total**	**752**,**643**	**70.8**	**892**

### Regional analysis: variation in oncological causes of death

**Fig. 2:**
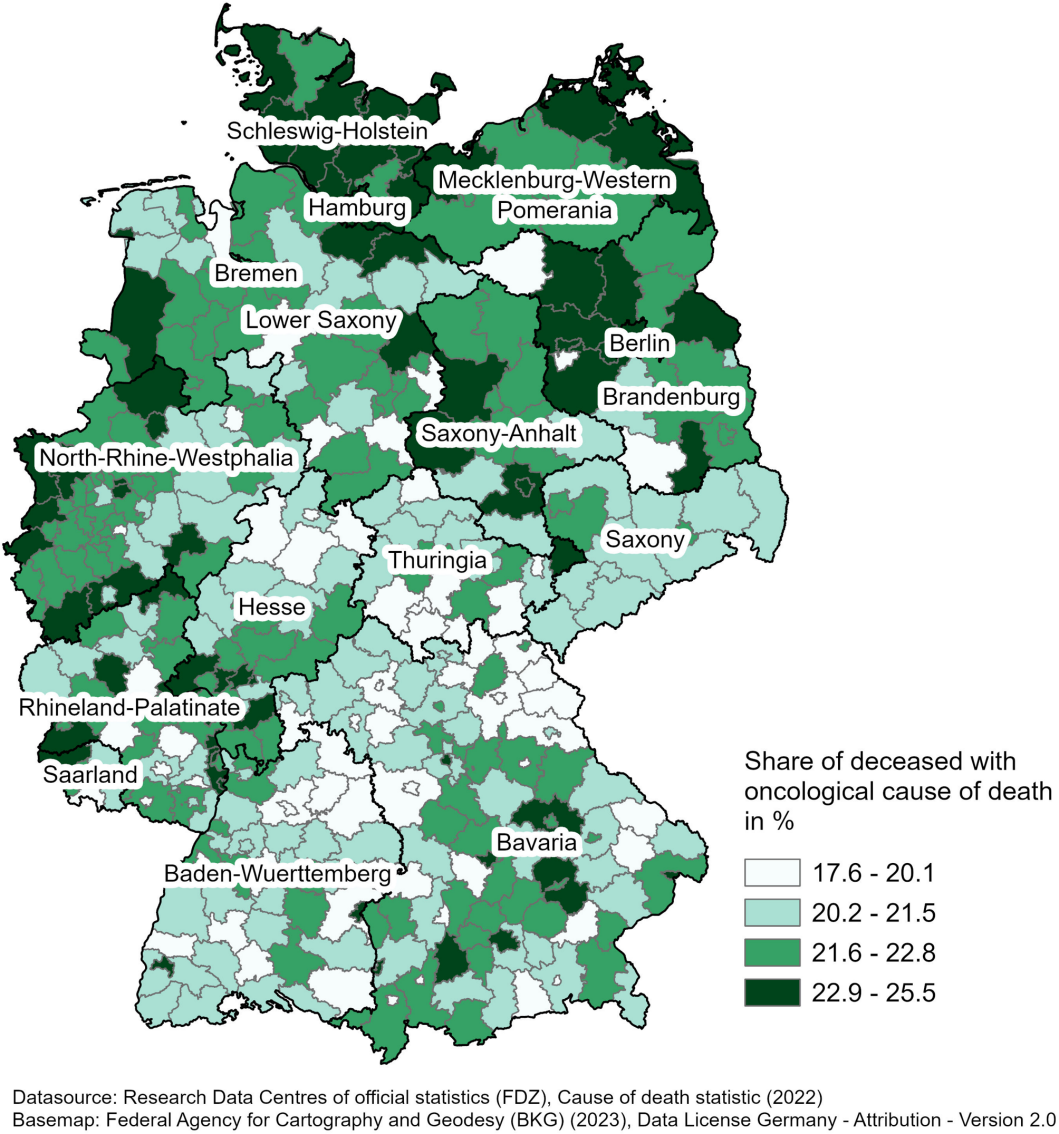
Share of deceased with oncological cause of death

Since oncological patients represent an important group in current palliative care provision, Fig. 2 illustrates the regional variation in deaths from oncological diseases, showing the proportion of deceased individuals whose cause of death is attributed to these conditions. This variation differs from the overall potential palliative care need, ranging from 17.6 to 25.5%. The highest proportions are observed in the federal states of Schleswig-Holstein and Saxony-Anhalt. These regions have values between 22.9 and 25.5%. The lowest percentages are in Rhineland-Palatinate and Bavaria, with values between 17.6% and 20.1%.

## Discussion

We present a first national analysis of the distribution of potential palliative care need in Germany on district level. The data on the numbers of causes of death from the year 2022 indicated a potential palliative care need for 70.8% of all deaths. This equates to approximately 892 individuals per 100,000 of the population per year. The number of individuals identified as potentially requiring palliative care ranged from 578 to 1,438 deceased per 100,000 inhabitants across the districts. The regional variation in potential palliative care need across Germany reflects demographic and socioeconomic disparities. With the method applied the potential palliative care need is higher in the eastern and some central federal states, while it tends to be lower in western and southern regions. In regions such as Saxony-Anhalt and some eastern areas, there is both a high potential need for palliative care and a high proportion of death due to oncological conditions. International studies underscore the influence of socioeconomic factors on palliative care. A Canadian study, for example, not only calculated the potential palliative care need but also the proportion of recipients of palliative care services, as ratio of the total number of deceased receiving palliative care over the total number of deceased with potential palliative care need. Results showed that this proportion is predicted by demographic and socioeconomic factors [9]. In Germany, the eastern and some parts of western regions are characterized by higher levels of socio-economic deprivation. In addition, the population in regions with high deprivation tend to higher smoking rates, lower levels of physical activity, and higher rates of obesity [25], which may contribute to the higher proportion of deaths from causes associated with potential palliative care need observed in these areas.

Moreover, our findings are slightly lower than results of a study conducted in Germany in 2013, in which the estimation, based on Murtagh’s refined method, indicated 78.0% of death cases potentially being eligible for palliative care [12]. This differs from the estimates for England, which indicate that 63% of all deceased individuals could have potentially benefited from palliative care [4]. Additionally, a recent German study displayed, that in 2019, the number of deceased with potential palliative care need identified was 15.6%-points smaller than the number identified based on the classification of Murtagh et al. using claims data as database. Compared to the Murtagh classification their specified ICD-10-based approach highlighted lower palliative care need in several disease-categories, particularly in kidney diseases, cardiovascular diseases, cerebrovascular diseases and cancer [26]. In a study by Brameld et al. in Western Australia, a different method was applied, considering people with at least one of ten conditions as amenable to palliative care (namely cancer, heart failure, renal failure, chronic obstructive pulmonary disease (COPD), Alzheimer’s disease, liver failure, Parkinson’s disease, motor neurone disease, HIV/AIDS and Huntington’s disease). They concluded that, depending on the sources of information used, between 43% and 73% of all deceased individuals had a condition that could potentially be treated with palliative care [6]. These discrepancies in estimations may be due to different observation years, trends in mortality or variations in age distribution as well as in data sources across countries.

Furthermore, our findings indicated that one-third of all deaths in Germany were attributed to diseases of the circulatory system. A comparison with the German study showed a slight decrease in the trend of mortality causes, with about 31% in 2013 and 34% in 2022 [12]. The mortality attributed to neoplasms declined over the years, although minor discrepancies in the ICD-10 code definitions included across different databases have been observed. In 2013, the proportion of death caused by neoplasms was about 25%, whereas in 2022, it was about 22% [12]. These results are in line with the trend described by the Robert Koch Institute in 2024, according to which the most common causes of death are cardiovascular diseases and cancer, with rates in both groups declining. This tendency contributed to an increase in life expectancy [27]. But, the results of a study conducted by Etkind et al. indicated an increasing trend in palliative care need from 2006 to 2014, with a notable increase of 42.4%. The study highlighted that dementia and cancer would be the primary contributors to this rising need [28]. 

Our findings indicate differences in gender distribution of deceased with and without potential palliative care need. The proportion of deceased with potential need was higher among women compared to males, however, the differences were rather small. Moreover, we demonstrated a statistically significant difference in the age at time of death between those who potentially required palliative care and those who did not. These findings are in line with Morin et al. who found that irrespective of the estimation methods, higher age and female sex were independently associated with the chance of being in need of palliative care [5]. A potential explanation for the higher age could be that patients potentially requiring palliative care may have suffered from chronic illnesses or serious conditions with severe exacerbations who are usually ill for many months or years and often progress at a slower rate [29]. Furthermore, a recent systematic review indicated that being a woman is associated with an increased chance of accessing palliative care, including both generalist and specialist palliative care, in comparison to men [30]. It is notable that the proportion of deceased people with oncological conditions within the overall population of deceased individuals was relatively low, despite oncological condition being one of the primary condition typically associated with palliative care need [31]. This discrepancy may be indicative of a gap in the provision of palliative care for non-oncological conditions. Early integration of palliative care, including the provision of specialist palliative care services, but also the earlier engagement of general palliative care providers, such as general practitioners, who can initiate palliative support at an earlier stage of the patient’s illness, is essential for ensuring timely symptom management and improving quality of life [32, 33]. Taking into account regional variations in care planning can be one crucial instrument to ensure sufficient and high-quality palliative care for all patients, irrespective of their place of residence and diagnoses. A differentiated view of the potential palliative care need and the actual provision of palliative care could enable the optimal use of available resources and, at the same time, would ensure that all individuals who need this care receive timely and appropriate support.

Some strengths and limitations of this study have to be mentioned: The main strength is that this study represents the first attempt to examine the specific palliative care need on a small-area level in Germany. It makes use of a comprehensive database, which includes access to official statistics and ICD codes. The data set is complete for the entire population, and long-term trends can be depicted. 

As for limitations, we could only include the cause of death entered on the death certificate in the official statistics. Therefore, only one of the published literature-based approaches to palliative care can be used, as the data base for all other methods are not available in Germany (e.g. comorbidities, data on hospital admissions). Future studies should consider using additional data sources such as primary care databases or hospital data.

Furthermore, causes of death are not an explicit indicator for actual palliative care need since they are based on diagnoses only. Palliative care need are determined by more complex and interacting factors than diagnoses alone. We can therefore not draw conclusions on either actual need as well as actual palliative care provided. These factors also depend on other factors, such as the local availability of palliative care services. Accessibility to the different palliative care services also varies on a regional level, showing that almost half a million people (for palliative care units), more than 10 Mio people (for palliative care advisory teams) and almost 250,000 people (for specialist palliative home care teams) live more than an hour away from their next service [34]. 

Moreover, it is crucial to consider that these estimates refer to the overall need for palliative care, regardless of the level of care or care setting. It should be understood as a comprehensive need that encompasses the full range of palliative care, from generalist to specialist settings. Population-based estimates based on causes of death statistics do not differentiate between types of care, as comprehensive information on clinical situation of the deceased is missing [5]. The distinction between generalist and specialist palliative care is of particular interest to policy makers and stakeholders, as the individual need for generalist or specialist care can vary over time and disease trajectory. Estimates that distinguish between the need for generalist and specialist palliative care are relevant to health care systems, also to address future need of care. Whereas there is regulated regional planning for hospital care and ambulatory physicians [35], the organization of palliative care services does not follow the same regional planning but is rather organized through networks and regional structures.

A further significant limitation that must be considered relates to the validity of the documentation of the causes of death on death certificates, with particular reference to ill-defined causes of death. The documentation of unknown causes of death (ICD-10 codes R00-R99), in our data 3.8%, might be problematic as this means that the actual underlying disease responsible for the death remains hidden. The share of this so called ill-defined causes of death differs across federal states [21]. Since 2011, dementia has been assigned a higher priority than other diseases listed on death certificates that qualify as underlying conditions for coding, there has been a decline in the number of deaths, particularly those from cardiovascular diseases associated with advanced age, such as heart failure and stroke [21]. Completed and properly completed death certificates form the basis for analyses that can contribute to improving healthcare. If certificates are not completed in accordance with the guidelines, the software IRIS can be used to adapt them accordingly [22]. Also, we show data aggregated on district level. Districts vary in population size (between approx. 35.000 and 1.9 million inhabitants) and population density (between 35 and 4900 inhabitants per square kilometer, all numbers based on 2023 data) [36]. Comparison of the impact of results on the different regions therefore needs to be done under this consideration.

## Conclusion

In conclusion, this study presents the first national analysis of potential palliative care need in Germany at district level. While these findings are slightly lower than those in the past, they are consistent with international estimates, such as those from England and Western Australia. The study highlights differences in palliative care need across gender, age, and cause of death, indicating that potential palliative care is especially needed, beyond those for individuals with oncological conditions, particularly elderly and individuals with chronic or non-oncological conditions. To face the rising need of palliative care in the coming decades, this analysis can serve as a basis for regional need. Since we analyzed only the potential need at the end of life based on death records, future studies should complement this by including longitudinal data on actual care utilization, local resources of palliative care, and a distinction between generalist and specialist palliative care provision. The local structures and accessibility also need to be addressed. Mixed methods studies based on routine data and primary data (both quantitative and qualitative) would be best to further address this important future challenge in health care provision at the end of life.

## Data Availability

The database was provided by the Federal Statistical Office.

## Declarations

The following statements should be included under the heading “Statements and Declarations” for inclusion in the published paper. Please note that submissions that do not include relevant declarations will be returned as incomplete.

## References

[1] World Health Organization (2025) Palliative care. https://www.who.int/health-topics/palliative-care Accessed 2025

[2] Radbruch L, Payne S (2009) White paper on standards and norms for hospice and palliative care in europe: part 1. Eur J Palliat Care 16(6):278–289

[3] Leitlinienprogramm O (2020) Erweiterte S3-Leitlinie palliativmedizin für patienten Mit einer nicht-heilbaren krebserkrankung. Langversion 2.2. https://www.dgpalliativmedizin.de/images/stories/pdf/LL_Palliativmedizin_Langversion_2.2.pdf Accessed 2024.

[4] Murtagh FE, Bausewein C, Verne J, Groeneveld EI, Kaloki YE, Higginson IJ (2014) How many people need palliative care? A study developing and comparing methods for population-based estimates. Palliat Med 28(1):49–58. 10.1177/0269216313489367 23695827 10.1177/0269216313489367

[5] Morin L, Aubry R, Frova L, MacLeod R, Wilson DM, Loucka M et al (2017) Estimating the need for palliative care at the population level: A cross-national study in 12 countries. Palliat Med 31(6):526–536. 10.1177/0269216316671280 27683475 10.1177/0269216316671280

[6] Brameld K, Spilsbury K, Rosenwax L, Murray K, Semmens J (2017) Issues using linkage of hospital records and death certificate data to determine the size of a potential palliative care population. Palliat Med 31(6):537–543. 10.1177/0269216316673550 27777376 10.1177/0269216316673550 PMC5405828

[7] Rosenwax LK, McNamara B, Blackmore AM, Holman CD (2005) Estimating the size of a potential palliative care population. Palliat Med 19(7):556–562. 10.1191/0269216305pm1067oa 16295289 10.1191/0269216305pm1067oa

[8] Borgsteede SD, Deliens L, Francke AL, Stalman WA, Willems DL, van Eijk JT et al (2006) Defining the patient population: one of the problems for palliative care research. Palliat Med 20(2):63–68. 10.1191/0269216306pm1112oa 16613401 10.1191/0269216306pm1112oa

[9] Panarella M, Saarela O, Esensoy AV, Jakda A, Liu ZA (2019) Regional variation in palliative care receipt in ontario, Canada. J Palliat Med 22(11):1370–1377. 10.1089/jpm.2018.0573 31090480 10.1089/jpm.2018.0573

[10] Higginson I (1997) Health care needs assessment: palliative and terminal care. In: Stevens A, Raftery J (eds) Health care needs assessment. Radcliffe Medical, Oxford, pp 183–260

[11] Gómez-Batiste X, Martínez-Muñoz M, Blay C, Espinosa J, Contel JC, Ledesma A (2012) Identifying needs and improving palliative care of chronically ill patients: a community-oriented, population-based, public-health approach. Curr Opin Support Palliat Care 6(3):371–378. 10.1097/SPC.0b013e328356aaed 22801465 10.1097/SPC.0b013e328356aaed

[12] Scholten N, Gunther AL, Pfaff H, Karbach U (2016) The size of the population potentially in need of palliative care in Germany - an Estimation based on death registration data. BMC Palliat Care 15(1):29. 10.1186/s12904-016-0099-2 26957121 10.1186/s12904-016-0099-2 PMC4782573

[13] Chukwusa E, Yu P, Verne J, Taylor R, Higginson IJ, Wei G (2020) Regional variations in geographic access to inpatient hospices and place of death: A Population-based study in england, UK. PLoS ONE 15(4):e0231666 32302344 10.1371/journal.pone.0231666 PMC7164606

[14] Chukwusa E, Verne J, Polato G, Taylor R, Higginson IJ, Gao W (2019) Urban and rural differences in geographical accessibility to inpatient palliative and end-of-life (PEoLC) facilities and place of death: a National population-based study in england, UK. Int J Health Geogr 18(1):8 31060555 10.1186/s12942-019-0172-1 PMC6503436

[15] Cohen J, Houttekier D, Onwuteaka-Philipsen B, Miccinesi G, Addington-Hall J, Kaasa S et al (2010) Which patients with cancer die at home? A study of six European countries using death certificate data. J Clin Oncol 28(13):2267–2273 20351336 10.1200/JCO.2009.23.2850

[16] Benchimol EI, Smeeth L, Guttmann A, Harron K, Moher D, Petersen I et al (2015) The reporting of studies conducted using observational Routinely-collected health data (RECORD) statement. PLoS Med 12(10):e1001885 26440803 10.1371/journal.pmed.1001885 PMC4595218

[17] Bundesamt für Kartographie und G (2022) Verwaltungsgebiete der Bundesrepublik Deutschland 1: 2.500. https://gdz.bkg.bund.de/index.php/default/digitale-geodaten/verwaltungsgebiete.html?___store=default Accessed 2024

[18] Statistische Ämter des Bundes und der Länder (2022) Fläche und Bevölkerung nach Ländern. https://www.statistikportal.de/de/bevoelkerung/flaeche-und-bevoelkerung Accessed 2024

[19] Statistisches Bundesamt (Destatis) (2019) Statistisches Jahrbuch 2019

[20] World Health Organization (2025) International Classification of Diseases (ICD) - Cause of death. https://www.who.int/standards/classifications/classification-of-diseases/cause-of-death Accessed 2025

[21] Stolpe S, Kowall B (2025) Todesursachenstatistik– wie fehlinterpretationen von mortalitätsdaten Vermieden Werden. Bundesgesundheitsblatt - Gesundheitsforschung - Gesundheitsschutz 68(2):167–175. 10.1007/s00103-024-03986-3 39630243 10.1007/s00103-024-03986-3 PMC11775058

[22] Federal Institute for Drugs and Medical Devices (2025) Iris software. https://www.bfarm.de/EN/Code-systems/Collaboration-and-projects/Iris-Institute/Iris-software/_node.html Accessed 2025

[23] Statistische Ämter des Bundes (2022) und der Länder: Todesursachenstatistik. https://www.regionalstatistik.de/genesis/online?operation=statistic&levelindex=0&levelid=1708504673419&code=23211#abreadcrumb Accessed 2024.

[24] Bundesinstitut für Arzneimittel und Medizinprodukte (2022) ICD-10-WHO - Internationale statistische Klassifikation der Krankheiten und verwandter Gesundheitsprobleme der WHO. https://www.bfarm.de/DE/Kodiersysteme/Klassifikationen/ICD/ICD-10-WHO/_node.html Accessed 2024

[25] Kroll LE, Schumann M, Hoebel J, Lampert T (2017) Regional health differences– developing a socioeconomic deprivation index for Germany. Robert Koch-Institut, Epidemiologie und Gesundheitsberichterstattung

[26] Slotina E, Ditscheid B, Meissner F, Marschall U, Wedding U, Freytag A (2024) A refined ICD-10 diagnoses-based approach for retrospective analysis of potential palliative care need and coverage in claims data of deceased. SAGE Open Med 12:20503121241269599. 10.1177/20503121241269599 39144525 10.1177/20503121241269599 PMC11322944

[27] Robert Koch Institut (2024) Mortality and causes of death. https://www.rki.de/EN/Topics/Noncommunicable-diseases/Health-in-the-course-of-life/Mortality-causes-of-death/mortality-and-causes-of-death-node.html Accessed 2025.

[28] Etkind SN, Bone AE, Gomes B, Lovell N, Evans CJ, Higginson IJ et al (2017) How many people will need palliative care in 2040? Past trends, future projections and implications for services. BMC Med 15(1):102. 10.1186/s12916-017-0860-2 28514961 10.1186/s12916-017-0860-2 PMC5436458

[29] Murray SA, Kendall M, Boyd K, Sheikh A (2005) Illness trajectories and palliative care. BMJ (Clinical Res ed) 330(7498):1007–1011. 10.1136/bmj.330.7498.1007 10.1136/bmj.330.7498.1007 PMC557152 15860828

[30] Rodríguez-Gómez M, Pastor-Moreno G, Ruiz-Pérez I, Escribà-Agüir V, Benítez-Hidalgo V (2024) Age- and gender-based social inequalities in palliative care for cancer patients: a systematic literature review. Front Public Health 12:1421940. 10.3389/fpubh.2024.1421940 39296836 10.3389/fpubh.2024.1421940 PMC11408182

[31] Schoenherr LA, Bischoff KE, Marks AK, O’Riordan DL, Pantilat SZ (2019) Trends in Hospital-Based specialty palliative care in the united States from 2013 to 2017. JAMA Netw Open 2(12):e1917043. 10.1001/jamanetworkopen.2019.17043 31808926 10.1001/jamanetworkopen.2019.17043 PMC6902777

[32] Temel JS, Greer JA, Muzikansky A, Gallagher ER, Admane S, Jackson VA et al (2010) Early palliative care for patients with metastatic non–small-cell lung cancer. N Engl J Med 363(8):733–742 20818875 10.1056/NEJMoa1000678

[33] Dalgaard KM, Bergenholtz H, Nielsen ME, Timm H (2014) Early integration of palliative care in hospitals: A systematic review on methods, barriers, and outcome. Palliat Supportive Care 12(6):495–513. 10.1017/S1478951513001338 10.1017/S1478951513001338 24621947

[34] Gesell D, Hodiamont F, Bausewein C, Koller D (2023) Accessibility to specialist palliative care services in germany: a geographical network analysis. BMC Health Serv Res 23(1):786. 10.1186/s12913-023-09751-7 37488579 10.1186/s12913-023-09751-7 PMC10364400

[35] Blümel M, Spranger A, Achstetter K, Maresso A, Busse R, Germany (2020) Health Syst Rev Health Syst Transit 22(6):1–272 34232120

[36] Destatis (2023) Kreisfreie Städte und Landkreise nach Fläche, Bevölkerung und Bevölkerungsdichte am 31.12.2023. https://www.destatis.de/DE/Themen/Laender-Regionen/Regionales/Gemeindeverzeichnis/Administrativ/04-kreise.html Accessed 2025

