# Correction: Patients potentially in need for palliative care in Germany—A regional small-area estimation based on death registry data

**DOI:** 10.1007/s43999-025-00072-2

**Published:** 2025-08-20

**Authors:** Daniela Gesell, Farina Hodiamont, Claudia Bausewein, Eva Grill, Daniela Koller

**Affiliations:** 1 Institute of Medical Data Processing, Biometrics and Epidemiology (IBE), Faculty of Medicine, LMU Munich, Munich, Germany; 2 Department of Palliative Medicine, LMU University Hospital, LMU Munich, Munich, Germany; 3 German Center for Vertigo and Balance Disorders (DSGZ), LMU University Hospital, LMU Munich, Munich, Germany

Following the publication of the original article [1], the authors would like to add a conflict of interest statement in the declarations. The statement is given below.

Conflict of interest

The author Daniela Koller is an Editor-in-Chief of “Research in Health Services & Regions”. She was not involved in the peer-review and handling of the manuscript. The authors declare no other financial or non-financial interests that are directly or indirectly related to the work submitted for publication.

The original article [1] has been corrected.
